# Perceived Social Support Attenuates the Association between Stress and Health-Related Quality of Life among Adults Experiencing Homelessness

**DOI:** 10.3390/ijerph182010713

**Published:** 2021-10-13

**Authors:** Midhat Z. Jafry, Jayda Martinez, Tzuan A. Chen, Michael S. Businelle, Darla E. Kendzor, Lorraine R. Reitzel

**Affiliations:** 1 Department of Biology and Biochemistry, College of Natural Sciences & Mathematics, University of Houston, Science & Research Building 2, 3455 Cullen Blvd Room 342, Houston, TX 77204, USA; mzjafry@central.uh.edu; 2 Department of Psychological, Health, and Learning Services, College of Education, University of Houston, 491 Farish Hall, Houston, TX 77204, USA; jamart58@central.uh.edu (J.M.); tchen3@central.uh.edu (T.A.C.); 3 HEALTH Research Institute, University of Houston, 1100 Health 2, 4849 Calhoun Rd., Houston, TX 77204, USA; michael-businelle@ouhsc.edu; 4 TSET Health Promotion Research Center, The University of Oklahoma Health Sciences Center, University of Oklahoma, 655 Research Parkway, Suite 400, Oklahoma City, OK 73104, USA; darla-kendzor@ouhsc.edu

**Keywords:** health-related quality of life, social support, perceived stress, homeless, health disparities

## Abstract

Health-related quality of life (HRQoL) is defined as a multidimensional subjective assessment of one’s physical and mental health. Homelessness is associated with numerous stressors that can reduce HRQoL. Social support is defined as the availability of individuals, or resources provided by individuals, to cope with stress. Interpersonal social support may be important in buffering HRQoL from the negative implications of stress. Here, we examine this association in a marginalized group known for high rates of physical and mental health comorbidities: adults experiencing homelessness. Participants (*N* = 581; 63.7% men; M_age_ = 43.6 ± 12.2) were recruited from homeless-serving agencies in Oklahoma City. Social support was measured with the 12-item Interpersonal Support Evaluation List (ISEL). HRQoL was measured by the Behavioral Risk Factor Surveillance System (BRFSS) survey using self-rated health, the number of poor mental and poor physical health days over the preceding 30 days, as well as the number of limited activity days as the result of poor mental and/or physical health. Perceived stress was assessed using the 4-item Perceived Stress Scale (PSS). The potential moderation effect of social support was examined by assessing the interaction term of social support and stress in a series of linear regression analyses, controlling for sex, age, months homeless, race, education, health insurance status, serious mental illness diagnosis, and recruitment agency/site. There was a significant interaction effect of social support and stress on the prediction of days of poor physical health, days of poor mental health, and days of limited activity (*p* in all cases ≤ 0.05). Results add to a growing literature on the potentially protective benefits of social support for HRQoL, extend them to a large sample of adults experiencing homelessness in the South, and demonstrate the significance of this moderating effect of social support over and above the influence of several prominent sociodemographic and diagnostic variables. Future work should determine if interventions designed to enhance social support can buffer HRQoL from the deleterious effects of stress among this vulnerable population.

## 1. Introduction

The U.S. Department of Housing and Urban Development (HUD) reported an estimated 580,000 individuals experiencing homelessness in the United States in 2020, with almost 4 in 10 of those individuals living unsheltered [1]. Adults experiencing homelessness face numerous stressors, such as a lack of health insurance and visual impairment [2], and have high rates of behavioral health issues that are associated with increased risks of premature morbidity and mortality [3,4,5,6,7,8,9]. Notable stress-inducing factors related to homelessness include exposure to violence [10]; food insecurity [11]; fear and mistrust [12]; lack of access to preventive medicine, engendering high use of emergency services [13]; criminal justice involvement [14]; poor sleep [15,16]; and discrimination [17], among others [11,18]. In fact, adults experiencing homelessness are known to endure various physical illnesses and chronic conditions that tend not to arise in the domiciled population until about 5–20 years later [19]. Longitudinal studies further reveal that individuals experiencing both homelessness and mental health disorders or infectious diseases are substantially less likely to receive primary or specialized care services, which can exacerbate present health conditions [20]. Consequently, this group tends to have lower health-related quality of life (HRQoL), a multi-dimensional subjective approximation of one’s own physical and mental health, relative to their domiciled counterparts [21,22].

Social support, or the availability of individuals, or resources provided by individuals, to cope with stress has been identified as a resiliency factor with the potential to improve health-related outcomes [23,24,25]. Furthermore, strong levels of social support have been associated with increased resilience and reduced stress within a sample of individuals experiencing homelessness [26]. In addition to lowering levels of stress, high levels of social support have been associated with reduced homelessness episodes, whereas low levels of social support have been associated with increased risks of repeated homelessness [27,28]. Thus, social support may be a crucial determinant in mitigating the effects of the numerous stressors linked to homelessness and their association with poorer HRQoL.

Previous research has revealed that older adults experiencing homelessness with high levels of emotional distress and low social support are more susceptible to poor HRQoL [29]. According to the postulates of the stress buffering model, social support is theorized to reduce detrimental impacts of perceived stress on health [30]. Extant studies have corroborated this model and reflect social support’s buffering role in counteracting the negative health implications of stress [31,32]. While the current literature has a handful of studies examining social support in the context of stress and health, most studies focus on a subset or specialized group (e.g., homeless youth) [31], homeless mothers [33], homeless smokers [29], and individuals with mental illnesses [26], among other groups, which may lead to results that are not generalizable to the larger population of adults experiencing homelessness.

The current study aims to address gaps in literature by evaluating the moderating role of social support in the association between stress and HRQoL, a comprehensive measure of physical and mental health, in a convenience sample of adults experiencing homelessness from several recruitment sites in Oklahoma City, OK. Given the multiple debilitating effects of homelessness, further research is necessary to better understand resiliency factors that can be considered in interventions to increase HRQoL among this group. Findings from the current study may have implications for future health policy and interventional strategies for this extremely marginalized population.

## 2. Materials and Methods

### 2.1. Participants

Participants represented a convenience sample of adults experiencing homelessness who were recruited from among 6 homeless-serving agencies and/or shelters in Oklahoma City, OK, in the summer of 2016. Recruitment was accomplished via posted fliers at the recruitment sites advertising a study meant to better understand health and health-related needs of individuals experiencing homelessness. Eligibility criteria were individuals ≥ 18 years of age, receiving services from ≥1 of the recruitment sites, and with >6th grade English literacy level as indicated by a score ≥ 4 on the Rapid Estimate of Adult Literacy in Medicine-Short Form [34]. A maximum possible enrollment of 800 adults was based on funding available for participants’ remuneration and the a priori duration of time that participating agencies allowed on-site data collection. Overall, 648 adults indicated interest in participating in this study, of which 38 were ineligible due to low literacy. Following eligibility verification, the study staff checked to ensure individuals only enrolled in the study once across the various sites. Eligible individuals provided informed consent for participation. Of the 610 enrolled participants, 29 used services from the recruiting agencies but indicated they had a residence, resulting in an analyzable sample of 581 adults.

### 2.2. Procedures

This study was approved by the Institutional Review Boards at the Universities of Houston and Oklahoma Health Sciences Center. Questionnaires were completed by participants on a computer where survey items were visible on the screen and read out loud to the participants over headphones. Study personnel were available for assistance as needed. Remuneration for participation was provided in the form of a $20 department store gift card.

### 2.3. Measures

#### 2.3.1. Participant Characteristics

Information on age, sex, race (Black/African American, White, Asian, Native America/Alaska Native, or multi-racial/other), education, health insurance status (some vs. none), lifetime homelessness (in months), and history of serious mental illness diagnosis (schizophrenia, depression, anxiety, post-traumatic stress, or bipolar disorders) was collected via self-report. The recruitment site was noted in study records and was included as a covariate in analysis.

#### 2.3.2. Social Support

Perceived social support was assessed using the 12-item Interpersonal Support Evaluation List [35]. Items were rated on a 4-point scale (potential range = 12–48), with higher scores indicating greater perceived social support. Cronbach’s alpha in this sample was 0.89.

#### 2.3.3. Perceived Stress

Perceived stress was assessed using the 4-item Perceived Stress Scale [36]. Items were rated on a 5-point scale (potential range = 0–16) to reflect perceived stress over the prior week, with higher scores indicating greater perceived stress. Cronbach’s alpha in this sample was 0.63.

#### 2.3.4. Health-Related Quality of Life

HRQoL was measured using 4 items from the Center of Disease Control and Prevention’s Behavioral Risk Factor Surveillance System survey [37]: (1) self-rated health (1 = excellent, 2 = very good, 3 = good, 4 = fair, 5 = poor), (2) poor physical health days (“Now thinking about your physical health, which includes physical illness and injury, for how many days during the past 30 days was your physical health not good?”), (3) poor mental health days (“Now thinking about your mental health, which includes stress, depression, and problems with emotions, for how many days during the past 30 days was your mental health not good?”), and (4) activity-limited days due to poor physical or mental health (potential range 0–30). This assessment expands beyond the general evaluation of individual and population health to reveal the impact of quality of life on health and the evident burden of chronic health conditions [38]. This measure is based on self-perceptions, and high scores on items are a significant predictor of mortality [39,40].

### 2.4. Data Analysis

**Figure 1:**
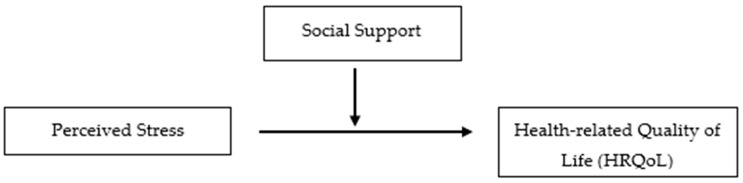
Moderation model of social support’s relation to perceived stress and health-related quality of life. The stress buffering model provides a conceptual framework for identifying a moderator of the association between stress and health-related quality of life. The independent variable is perceived stress. The dependent variable is HRQoL, measured by self-rated health, the number of poor physical and mental days, and activity limited due to poor physical/mental days. Social support is the hypothesized moderator that lies between stress and HRQoL. The stress buffering model would suggest that social support would buffer a presumed negative association between stress and HRQoL.

Participant descriptive data and intercorrelations between variables were examined. To assess the moderation model (Figure 1), the interaction term of social support and stress was included in a series of linear regressions adjusted for age, sex, race, education, health insurance status, lifetime homelessness, any history of serious mental illness, and recruitment site, predicting each respective HRQoL variable (self-rated health, poor physical health days, poor mental health days, and limited activity days in the past month). Continuous variables were mean centered prior to analysis. Interaction effects were graphed using the pick-a-point approach, where the mean and the mean ± 1 standard deviation (SD) were calculated to represent high, moderate, and low levels of social support. The Johnson–Neyman technique [41] was used to probe significant interactions within the observed range of the moderator. Significance was set at *p* ≤ 0.05. All analyses were conducted using SAS 9.4 (Cary, NC, USA).

## 3. Results

### 3.1. Sample Descriptives

Participants (*N* = 581; 63.68% men; M_age_ = 43.64 ± 12.16) were homeless for an average of 42.36 ± 51.25 months over their lifetimes. Overall, 10.69% of the participants rated their health as excellent, 20.17% as very good, 32.76% as good, 27.59% as fair, and 8.79% as poor. Participants reported 7.95 ± 10.69 poor physical health days, 10.78 ± 11.8 poor mental health days, and 6.36 ± 9.59 limited activity days over the previous month (Table 1).

Correlation analyses indicated significantly positive associations between having a history of major mental health disorder diagnosis and poorer HRQoL (*r* = 0.166–0.345; *ps* < 0.001) and perceived stress and poorer HRQoL (*r* = 0.229–0.479; *ps* < 0.001). Additionally, less perceived social support was associated with poorer HRQoL (*r* = −0.345–−0.189; *ps* < 0.001). See Table 2.

### 3.2. Moderation Analyses

#### 3.2.1. Self-Rated Health

**Figure 2:**
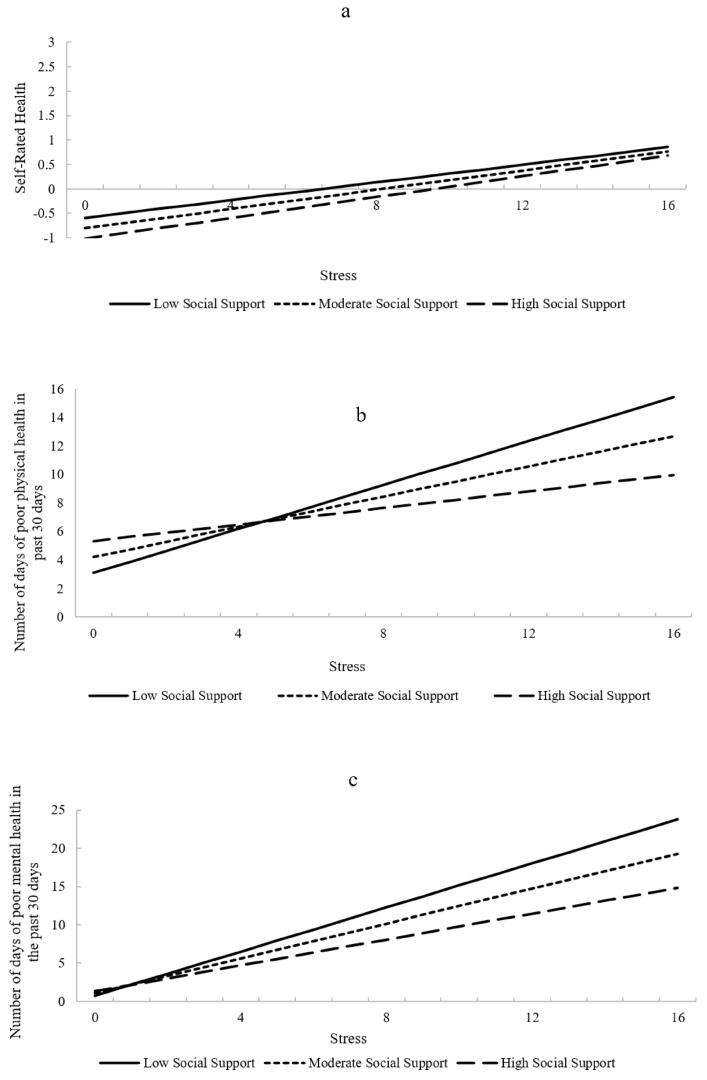

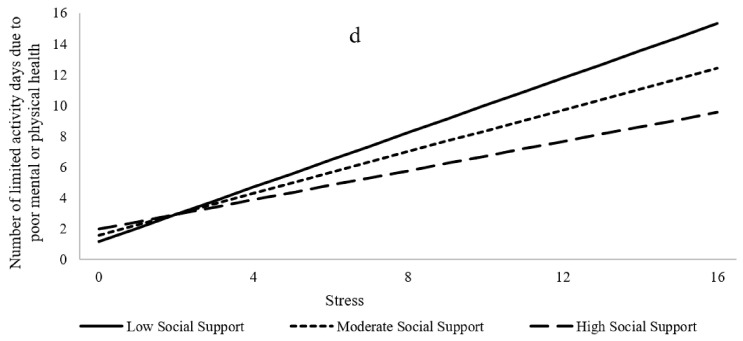
Moderating effect of social support on the association between stress and health-related quality of life among adults experiencing homelessness (*N* = 581). Covariates were age, sex, race, education, health insurance status, lifetime homelessness, a history of serious mental illness, and recruitment site. (**a**) shows moderation of social support in the association between stress and self-rated health. (**b**) shows moderation of social support in the association between stress and number of days of poor physical health. (**c**) shows moderation of social support in the association between stress and number of days of poor mental health. (**d**) shows moderation of social support in the association between stress and number of activity-limited days due to poor physical or mental health.

Social support was not a significant moderator of the association between stress and self-rated health (*p* = 0.466), indicating that the relationship between stress and self-rated health does not vary at different levels of social support. The moderation effect was also non-significant when assessed as a binary variable (excellent/very good/good versus fair/poor (*p* = 0.092)) (Table 3; Figure 2a).

#### 3.2.2. Poor Physical Health Days

There was a significant interaction effect of social support and stress on the prediction of days of poor physical health (B = −0.028; *p* = 0.03) (Table 3). Simple slopes analyses revealed that stress was significantly related to a greater number of poor physical health days at low and moderate, but not high, levels of social support (mean, ±1 SD; low = 24.19 (B = 0.77; *p* < 0.001), moderate = 32.91 (B = 0.53; *p* = 0.0001), and high = 41.64 (B = 0.29; *p* = 0.0975)) (Figure 2b). The Johnson–Neyman technique revealed that the significant point on the continuum of the moderator social support (PSS; not mean centered) was 40.38, meaning stress was significantly associated with poor physical health days in the preceding month with social support scores ≤ 40.38 (77.5% of the sample).

#### 3.2.3. Poor Mental Health Days

There was a significant interaction effect of social support and stress on the prediction of days of poor mental health (B = −0.034; *p* = 0.007) (Table 3). Simple slopes analyses revealed that perceived stress was significantly related to poor mental health days at low, moderate, and high levels of social support (mean, ±1 SD; low = 24.19 (B = 1.44; *p* < 0.001), moderate = 32.91 (B = 1.14; *p* < 0.001), and high = 41.64 (B = 0.84; *p* < 0.001)) (Figure 2c). However, the Johnson–Neyman technique revealed that there was no statistically significant transition point within the possible ranges of social support, which indicates that higher social support weakened the positive relationship between stress and the number of days of poor mental health in the preceding month (shown as a positive correlation between perceived stress and days experiencing poor mental health in the preceding 30 days) but increased social support did not make the significant relationship disappear at any observed level of perceived stress.

#### 3.2.4. Activity-Limited Days Due to Poor Physical or Mental Health

There was a significant interaction effect of social support and stress on the prediction of activity-limited days due to poor physical and mental health (B = −0.024; *p* = 0.033) (Table 3). Simple slopes analyses revealed that perceived stress was significantly related to activity-limited days at low, moderate, and high levels of social support (mean, ±1 SD; low = 24.19 (B = 0.89; *p* < 0.001), moderate = 32.91 (B = 0.68; *p* < 0.001), and high = 41.64 (B = 0.47; *p* = 0.002)) (Figure 2d). The Johnson–Neyman technique revealed that the significant point on the continuum of the social support (PSS; not mean centered) was 46.135, meaning perceived stress was significantly associated with activity-limited days due to poor physical or mental health, with social support scores ≤ 46.14 (94.55% of the sample).

## 4. Discussion

Individuals experiencing homelessness face an elevated risk of poor HRQoL based on numerous and significant daily and chronic stressors [21,22]. The current study supports the role of perceived social support in mitigating the positive association between stress and a greater number of poor physical health days, poor mental health days, and activity-limited days due to these factors. As suggested by the stress buffering model [33], our results show that greater perceived social support can attenuate the association between stress with days of functional impairment resulting from poor physical and mental health and this pattern can be extended to a sizeable sample of adults experiencing homelessness. Similar to results seen with domiciled adults [25,26,28,32], social support appears to function as a resiliency factor in buffering HRQoL from the deleterious effects of stress in this marginalized group. The current study was cross sectional; therefore, causal implications are untested and the potential for bidirectionality of associations exists; however, previous longitudinal studies have revealed that social support has protective benefits for HRQoL [42]. Although more research is needed, these findings may support the potential role of perceived social support in interventions to address several indicators of poor HRQoL experienced among homeless adults.

Individuals experiencing homelessness are at a very high risk for social exclusion, limited social support, and disruptions in social support [43,44]. Prior studies with domiciled adults have found that high social support is associated with increased resilience and reduced stress [29]; our results suggest similar relations may be applicable to homeless adults. Thus, despite the fact that this population is known to experience a disproportionate amount of stressors relative to the domiciled population [45] and exhibit high rates of mental and physical health conditions [3,4,5,6,7,8,9], interventions designed to foster social support for individuals experiencing homelessness may benefit their overall experience of physical and/or mental health. Consistent with a recovery-oriented approach to the care of individuals experiencing homelessness, results suggest the potential importance of assessing the capability of the multi-level ecological networks surrounding these adults to assist in their weathering of stressors so that any gaps can be addressed in holistic resiliency building [46]. Thus, interventions to bolster social support for this marginalized group can and should be considered across multiple levels, including those directed at individuals, agencies, and systems that are frequently encountered by this group. Support building efforts need not exclusively come from formal caregivers; rather, peer support programs may also be helpful for increasing resiliency and improving HRQoL [47]. Moreover, recognizing that social support can come in various forms (e.g., financial, practical, and emotional), it is imperative that individual deficits and personal needs be considered in intervention planning. Additionally, building social support is not merely about adding to the social network; research suggests that the quality of social relationships is important to health, particularly for homeless women, suggesting the importance of having low-conflict social relationships [44,48]. Finally, the effect of individual circumstances (e.g., living in sheltered versus unsheltered settings, transition to more permanent housing, or caregivers for children) should be considered in interventions to build and maintain social support for this population, knowing that circumstances, and a change in them, can be disruptive to extant support networks, requiring reassessment and additional resource/resiliency planning [49,50,51,52]. Ultimately, such efforts to build supportive social networks may have positive effects on mental and physical manifestations of poor HRQoL among individuals experiencing homelessness.

Notably, the present study yielded null results when assessing social support’s moderating effects on the association between stress and days of poor physical health at high levels of social support. This is contradictory to prior work that found that social support moderates the relationship between stress and poor physical health at all levels of social support in individuals with children who have disabilities [53]. Results may reflect differences in samples and assessment methods, as well as a lack of information on the quality of social support relative to the respective individual’s needs [54]. For example, it may be that at high levels of social support, the quality of the social support received moderates the association between stress and poor physical health days. Additional work is needed to better understand the implications of these findings; however, they suggest a limited role of social support, as assessed in this study, in the impact of stress on perceived days of poor physical health once it surpasses a certain, moderate point.

In the current study, social support was not a significant moderator of the association between perceived stress and self-reported health, although a trend toward significance was observed when it was examined as a binary variable. Nevertheless, our results suggest that improving social support may have no or a very limited role in addressing the effects of stress on self-rated health overall. Future work should examine other changeable factors that may more directly affect self-rated health. A prior study found that homeless women are more prone to reporting poor HRQoL as compared to men and social support is associated with self-rated health only in women [55]. Thus, social support may differentially affect the effect of stress on self-rated health based on sex; additional research is needed to more fully explore these possibilities and their impact on the need for sex-specific intervention approaches to improve self-rated health. Nonetheless, the current study addresses gaps in the literature by extending prior research on domiciled adults to adults experiencing homelessness and highlighting the potential moderating role of social support in the association between perceived stress and negative HRQoL in a large convenience sample of adults experiencing homelessness in Oklahoma City, OK, USA.

## 5. Limitations

Limitations include the cross-sectional nature of the study; causation cannot be inferred. Future research examining these factors in a longitudinal study is necessary to determine causation and confirm the promise of social-support-based interventions to impact stress and HRQoL. Additionally, the sample included only homeless adults; thus, the current study may not be representative of the experiences of homeless youth, pregnant/postpartum homeless mothers, etc. A point-in-time survey/count of individuals experiencing homelessness in Oklahoma City, OK, around the time of data collection indicated the following racial proportions: 58% White, 30% Black, 7% Native American, and 5% other/multi-races, with female representation at ~35% [56]. This compared with the present study as follows: ~54% White, ~20% Black, ~12% Native American, and ~14% other/multi-races, with female representation at ~36%. Thus, the convenience sample in this study may have been somewhat representative of the underlying population of homeless adults in the area, at least with regard to sex distribution and overall proportionality of racial representation. Additional limitations include that this study was conducted within a single urban area, with instrumentation provided in English only, requiring a 6th grade literacy level for enrollment. Therefore, results may not translate to the experiences of individuals experiencing homelessness in other cities and rural areas, those who are non-English speaking, or those who have limited literacy skills. Future studies should replicate results in other samples to develop a more comprehensive understanding of the potentially mediating role of social support in the relations between stress and HRQoL. Additionally, a longitudinal study may be advantageous in monitoring the effects of social support on the relationship between stress and HRQoL over time and further assessing if low-cost interventions can attenuate the relationship between stress and HRQoL.

## 6. Conclusions

Individuals experiencing homelessness are known to experience many stressors [10,11,12,13,14,15,16,17,18], limited social support [57], and poor HRQoL [21,22] relative to domiciled groups, ultimately increasing their susceptibility to premature morbidity and mortality [3,4,5,6,7,8,9]. Results of this cross-sectional study among adults experiencing homelessness suggest that social support moderates the association between stress and three of four components of HRQoL (poor physical health days, poor mental health days, and limited activity days due to poor mental or physical health) in models that included several prominent sociodemographic and diagnostic variables. Therefore, although further, longitudinal study is required, interventions to improve social support, particularly for those with low social support, may positively affect perceptions of the day-to-day effects of stress on physical and mental health functioning. It will be important to consider the dynamic individual, contextual, and systemic circumstances and needs in designing and implementing social-support-based interventions for adults within this marginalized group.

## Figures and Tables

**Table 1:** Sample characteristics (*N* = 581 adults experiencing homelessness).

Characteristics	n (SD)/% [*n*]
Sex	
Female	36.32 [211]
Age	43.64 (12.16)
Race	
White	53.65 [309]
Black or African American	19.62 [113]
Asian	0.35 [2]
Native American/Alaska Native	12.33 [71]
Multi-racial/Other	14.06 [81]
Years of Education	11.94 (2.04)
Insurance Status	
Insured	30 [174]
Serious Mental Illness	
Yes	65.69 [381]
Data Collection Site	
Site 1	41.31 [240]
Site 2	8.61 [50]
Site 3	30.29 [176]
Site 4	10.15 [59]
Site 5	7.23 [42]
Site 6	2.41 [14]
Lifetime Homeless (in months)	42.36 (51.25)
Social Support	32.91 (8.72)
Perceived Stress	7.67 (3.58)
Self-rated Health (Continuous)	3.04 (1.12)
Self-rated Health (Binary)	
Fair or Poor	36.38 [211]
Excellent, Very Good, or Good	63.62 [369]
Poor Physical Health Days (past 30 days)	7.95 (10.69)
Poor Physical Health Days (past 30 days)	
0	37.54 [217]
1–13	38.75 [224]
≥14	23.7 [137]
Poor Mental Health Days (past 30 days)	10.78 (11.80)
Poor Mental Health Days (past 30 days)	
0	32.18 [186]
1–13	31.66 [183]
≥14	36.16 [209]
Limited Activity Days (past 30 days)	6.36 (9.59)
Limited Activity Days (past 30 days)	
0	48.62 [281]
1–13	31.49 [182]
≥14	19.90 [115]

Note: Social support was measured with the 12-item Interpersonal Support Evaluation List; perceived stress was measured using the 4-item Perceived Stress Scale. HRQoL variables in the table were presented in multiple ways (e.g., continuous, as analyzed, but categorical for descriptive purposes.) Self-rated health (continuous) was coded as follows: 1 = excellent, 2 = very good, 3 = good, 4 = fair, and 5 = poor.

**Table 2:** Correlations between variables of interest among individuals experiencing homelessness in Oklahoma City, OK (*N* = 581).

	1	2	3	4	5	6	7	8	9	10	11
1. Sex (ref: Male)	1										
2. Age	−0.107 *	1									
3. Years of Education	0.041	0.084 *	1								
4. Any Insurance (Ref: No)	0.188 ***	0.040	0.039	1							
5. Serious Mental Illness (Ref: No)	0.159 ***	0.075	0.024	0.101 *	1						
6. Lifetime Homeless (in months)	−0.116 **	0.246 ***	−0.039	0.026	−0.004	1					
7. Social Support	0.069	−0.093 *	0.069	−0.066	−0.118 **	−0.085 *	1				
8. Perceived Stress	0.089 *	−0.035	−0.042	−0.054	0.200 ***	−0.047	−0.432 ***	1			
9. Poor Self-rated Health	0.113 **	0.208 ***	−0.002	0.012	0.166 ***	0.039	−0.290 ***	0.382 ***	1		
10. Poor Physical Health Days (past 30 days)	0.009	0.237 ***	0.055	−0.005	0.227 ***	0.093 *	−0.189 ***	0.229 ***	0.447 ***	1	
11. Poor Mental Health Days (past 30 days)	0.125 **	0.055	0.024	−0.029	0.345 ***	−0.001	−0.345 ***	0.479 ***	0.358 ***	0.538 ***	1
12. Limited Activity Days (past 30 days)	0.022	0.185 ***	0.0269	0.060	0.224 ***	0.060	−0.282 ***	0.330 ***	0.345 ***	0.563 ***	0.597 ***

Note: * *p* < 0.05; ** *p* < 0.01; *** *p* < 0.001. Social support was measured with the 12-item Interpersonal Support Evaluation List; perceived stress was measured using the 4-item Perceived Stress Scale.

**Table 3:** Linear regression model predicting health-related quality of life among individuals experiencing homelessness in Oklahoma City, OK (*N* = 581).

Outcomes of Interest	Key Variables for Interaction	Estimate	SE	*p*-Value
Self-rated Health	Social Support	−0.017	0.005	0.001
	Perceived Stress	0.099	0.014	<0.0001
	Social Support*Perceived Stress	0.001	0.001	0.466
Poor Physical Health Days	Social Support	−0.083	0.055	0.131
	Perceived Stress	0.529	0.137	<0.0001
	Social Support*Perceived Stress	−0.028	0.013	0.030
Poor Mental Health Days	Social Support	−0.228	0.054	<0.0001
	Perceived Stress	1.140	0.135	<0.0001
	Social Support*Perceived Stress	−0.034	0.012	0.007
Activity-Limited Days Due to Poor Physical or Mental Health	Social Support	−0.134	0.048	0.006
	Perceived Stress	0.679	0.120	<0.0001
	Social Support*Perceived Stress	−0.024	0.011	0.033

Note: SE: standard error. Covariates (not displayed in tables) included age, sex, race, education, health insurance status, lifetime homelessness, a history of serious mental illness, and recruitment site.

## Data Availability

The data presented in this study are available on request from the corresponding author. The data are not publicly available due to ethical restrictions based on informed consent agreements.
